# *Glossina* from the Republic of the Congo: species identification by MALDI-TOF MS and research of associated micro-organisms

**DOI:** 10.1051/parasite/2026007

**Published:** 2026-02-05

**Authors:** Irina Babakana Bemba, Zaina Amirat, Philippe Parola, Christophe Antonio Nkondjio, Arsene Lenga, Lionel Almeras, Adama Zan Diarra

**Affiliations:** 1 Marien Ngouabi University B.P. 69 Brazzaville Republic of the Congo; 2 Organisation de Coordination pour la lutte contre les Endémies en Afrique Centrale (OCEAC) B.P. 288 Yaoundé Cameroun; 3 Aix Marseille Univ, SSA, RITMES 13005 Marseille France; 4 IHU-Méditerranée Infection 13005 Marseille France; 5 Unité Parasitologie et Entomologie, Département Microbiologie et Maladies Infectieuses, Institut de Recherche Biomédicale des Armées 13005 Marseille France; 6 IRD, MINES, Maladies Infectieuses, Négligées et Émergentes au Sud 13005 Marseille France

**Keywords:** *Glossina*, Species identification, MALDI-TOF MS, *Trypanosoma*, Molecular biology

## Abstract

Human African trypanosomiasis (HAT) and Animal African trypanosomiasis (AAT), transmitted by *Glossina* species, remain major health and economic burdens in Africa. Accurate vector identification is essential for effective control strategies. However, current identification methods of *Glossina* species based on morphological and/or molecular techniques have several limitations that often hinder reliable species-level classification. This study assessed matrix-assisted laser desorption/ionization time-of-flight mass spectrometry (MALDI-TOF MS) as an alternative or complementary approach to morphological and molecular methods for *Glossina* species identification and explored its ability to detect infection status. A total of 265 tsetse flies were collected and morphologically classified into the *Glossina palpalis* group (*n* = 200) and the *Glossina fuscipes* group (*n* = 65), later confirmed by molecular analysis as *Glossina palpalis palpalis* and *Glossina fuscipes quanzensis*, respectively. Spectra were generated from wings, legs, and thoraxes to identify the most suitable body parts. *For G. p. palpalis*, high-quality spectra were obtained from wings (98.0%), legs (96.5%), and thoraxes (93.5%); for *G. f. quanzensis*, corresponding values were 89.2%, 87.7%, and 72.3%. Blind testing showed that 89.5% of spectra for *G. p. palpalis* and 95.2% for *G. f. quanzensis* matched morphological identification, with 87.0% and 94.6%, respectively, reaching relevant score thresholds. Molecular screening detected *Trypanosoma congolense* DNA in nine specimens, but MALDI-TOF MS spectra could not distinguish infected from uninfected flies. These findings demonstrate that MALDI-TOF MS is a rapid, reliable tool for *Glossina* species identification, particularly using wings and legs, but is unsuitable for infection status determination.

## Abstract (fr)

La trypanosomiase humaine africaine (THA) et la trypanosomiase animale africaine (TAA), transmises par des espèces de *Glossina*, constituent toujours un fardeau sanitaire et économique majeur en Afrique. L’identification précise du vecteur est essentielle à l’efficacité des stratégies de lutte. Cependant, les méthodes d’identification actuelles des espèces de *Glossina*, basées sur des techniques morphologiques et/ou moléculaires, présentent plusieurs limitations qui entravent souvent une classification fiable au niveau de l’espèce. Cette étude a évalué la spectrométrie de masse MALDI-TOF comme approche alternative ou complémentaire aux méthodes morphologiques et moléculaires pour l’identification des espèces de *Glossina* et a exploré sa capacité à détecter le statut infectieux. Au total, 265 mouches tsé-tsé ont été collectées et classées morphologiquement en deux groupes : *Glossina palpalis* (*n* = 200) et *Glossina fuscipes* (*n* = 65). L’identification de ces espèces a été confirmée ultérieurement par analyse moléculaire, respectivement comme *Glossina palpalis palpalis* et *Glossina fuscipes quanzensis*. Des spectres ont été générés à partir des ailes, des pattes et du thorax afin d’identifier les parties du corps les plus pertinentes. Pour *G. p. palpalis*, des spectres de haute qualité ont été obtenus à partir des ailes (98,0 %), des pattes (96,5 %) et du thorax (93,5 %). Pour *G. f. quanzensis*, les valeurs correspondantes étaient de 89,2 %, 87,7 % et 72,3 %. Des tests à l’aveugle ont montré que 89,5 % des spectres de *G. p. palpalis* et 95,2 % de ceux de *G. f. quanzensis* correspondaient à l’identification morphologique, avec respectivement 87,0 % et 94,6 % atteignant les seuils de score pertinents. Le criblage moléculaire a détecté l’ADN de *Trypanosoma congolense* dans neuf spécimens, mais les spectres MALDI-TOF MS n’ont pas permis de distinguer les mouches infectées des non infectées. Ces résultats démontrent que la spectrométrie de masse MALDI-TOF est un outil rapide et fiable pour l’identification des espèces de *Glossina*, notamment à partir des ailes et des pattes, mais qu’elle ne convient pas à la détermination du statut infectieux.

## Introduction

*Glossina*, commonly known as tsetse flies, are a genus of arthropod belonging to the family Glossinidae. Both males and females are obligate hematophagous feeders. *Glossina* is a single genus with 32 recognized species and subspecies [5, 60], many of which act as primary vectors of trypanosomes parasitic protozoa responsible for two major forms of trypanosomiasis: Human African Trypanosomiasis (HAT), also known as sleeping sickness, and Animal African Trypanosomiasis (AAT) [5, 60]. Human infections are fatal if left untreated; however, tools for controlling the disease remain limited. Moreover, reports of drug resistance are becoming increasingly common [62]. Efforts to develop vaccines against HAT have so far been unsuccessful, as reports have shown that treatments based on older trypanocides (melarsoprol, suramin, pentamidine) are associated with significant adverse side effects [4]. Moreover, reports of drug resistance are becoming increasingly common [4]. However, recent trials with a new trypanocide like acoziborole have demonstrated high efficacy and mild to moderate adverse effects, making acoziborole a promising treatment in efforts to achieve the WHO’s goal of interrupting HAT transmission by 2030 [12]. The populations most at risk of *Glossina* bites, and consequently these diseases, are those living in rural areas and involved in activities such as agriculture, fishing, livestock farming, or hunting [55].

In the Republic of the Congo (Congo-Brazzaville), 11 species of *Glossina* have been reported, including *Glossina* (*G.*) *frezili*, *G. fuscipes quanzensis*, *G. fuscipes fuscipes* Newst. 1910, *G. palpalis palpalis* Rob. Desv. 1830, *G.* (*Nemorhina*) *caligenea*, *G. tabaniformis*, *G. fusca congolensis*, *G. nashi*, *G. schwetzi*, *G. pallicera newsteadi*, *G. hamingtoni* [6]. Three of the *Glossina* species mentioned above are involved in the transmission of HAT across different regions of Africa: *G. fuscipes quanzensis* in Central Africa, including the Republic of the Congo [56], *G. fuscipes fuscipes* in East Africa [47], and *G. palpalis palpalis* in West Africa [21]. In the Republic of the Congo, the prevalence of HAT was historically relatively high, ranging from 5.5% to 10% during the 1980s and 1990s [6]. By 2019, it had declined significantly, with a national prevalence estimated at 0.1% [6]. In 2021, the country reported 425 HAT cases, accounting for more than half of the global cases that year [58]. However, transmission remains active in certain foci, particularly in the savannah areas and along the Congo River corridor, with Loudima, Ngabé, and Mpouya being the most affected localities [6]. The overall decrease in transmission is attributed to improved case detection and management, vector control measures, and better road infrastructure [6]. Nevertheless, a clearer understanding of the epidemiological situation in remote areas is still needed to strengthen eradication strategies and prevent disease resurgence. However, to date, no study has specifically addressed AAT in the country. Nevertheless, in a study conducted in three active HAT foci, 51.22% of the tested animals were found to carry Trypanosoma DNA, including *Trypanosoma congolense savannah* (67.2%) and *Trypanosoma brucei* (s.l.) (32.8%) [8].

Accurate identification of vector arthropod species is crucial for managing and preventing disease emergence, monitoring its spread, assessing transmission risk, and implementing effective control strategies [16]. *Glossina* species are typically identified using morphological characteristics or molecular biology techniques [26]. While both methods are effective, they present certain limitations. Morphological identification methods have several limitations, notably the requirement for taxonomic expertise, which may be insufficient to differentiate cryptic or closely related species or subspecies [26]. In addition, specimen integrity is critical for accurate classification, as the loss of essential morphological features can hinder proper identification [32, 57]. Molecular techniques also face several constraints, including the time-consuming nature of the procedures, the high cost of reagents, and the absence or limited availability of reference sequences for certain species in public databases such as GenBank (NCBI) [63]. For instance, only a partial sequence of the cytochrome c oxidase I (*COI*) gene is available for *G. tabaniformis*, and no genomic data are currently available for *G. longipalpis*. In some cases, this approach fails to discriminate between subspecies within a species complex, as observed with the *G. fuscipes* group, specifically *G. f. fuscipes* sensu stricto, *G. f. quanzensis*, and *G. f. martinii* when using the *COI* gene [26].

In the early 2010s, a proteomic approach known as matrix-assisted laser desorption/ionization time-of-flight mass spectrometry (MALDI-TOF MS) was proposed as an alternative method for identifying various arthropod families. Numerous studies have demonstrated the efficiency, speed, accuracy, and low cost of this technique in both medical and veterinary entomology [50, 51, 63]. Although MALDI-TOF MS has been widely applied for arthropod identification, only two studies to date have focused on its use for identifying *Glossina* species [33, 34]. The first study dedicated to the application of MALDI-TOF MS for the identification of *Glossina* species was conducted on laboratory-reared specimens, including *G. m. morsitans*, *G. austeni*, *G. pallidipes*, *G. palpalis gambiensis*, and *G. brevipalpis* [34]. Multiple body parts were analyzed: head, thorax, abdomen, wings, legs, and whole insects. The findings demonstrated the potential of MALDI-TOF MS for species-level identification of *Glossina*; however, identification accuracy varied depending on the specific spectral signatures of individual body parts and whole specimens. These results highlight the importance of analyzing multiple body parts to improve identification reliability. Despite the time required to analyze several body part from the same specimen by MS, its low cost for reagents, its rapidity and identification robustness remain factors that should not be neglected for quick, relevant species classification [34]. The second study evaluated the capacity of MALDI-TOF MS to discriminate *G. palpalis gambiensis* from *G. tachinoides*, based on specimens collected from two regions of Burkina Faso [33]. Only wings were used for analysis, with specimens stratified by sex and geographical origin. Cluster analysis indicated that while some grouping occurred by species and/or sex, specimens were most distinctly clustered according to their geographic origin [33].

Integrative taxonomy is an identification approach that combines morphological, molecular, proteomic, ecological, and behavioral data to achieve more accurate species identification and delimitation. This approach reduces misidentification errors and helps to reveal cryptic diversity that might be overlooked by traditional taxonomy [48]. In the case of *Glossina* spp., this approach is particularly important, as morphological similarities between species and subspecies can lead to misidentifications, and integrative taxonomy can help to clarify species boundaries and to enhance the reliability of surveillance programs [20, 26].

In a context where HAT and AAT remain major public health and veterinary challenges in the southern region of the Republic of the Congo, the accurate identification of *Glossina* species and detection of associated microorganisms are critical for the development and implementation of effective disease control strategies. This study therefore aimed to evaluate the applicability of MALDI-TOF MS for the identification of alcohol-preserved *Glossina* specimens and their associated microbial communities.

## Materials and methods

### Ethics

This study was approved by the ethics committee of CERSSA (*Comité d’Ethique de la Recherche en Sciences de la Santé*) under approval number 206/MRSIT/IRSSA/CERSSA.

### Study sites

**Figure 1:**
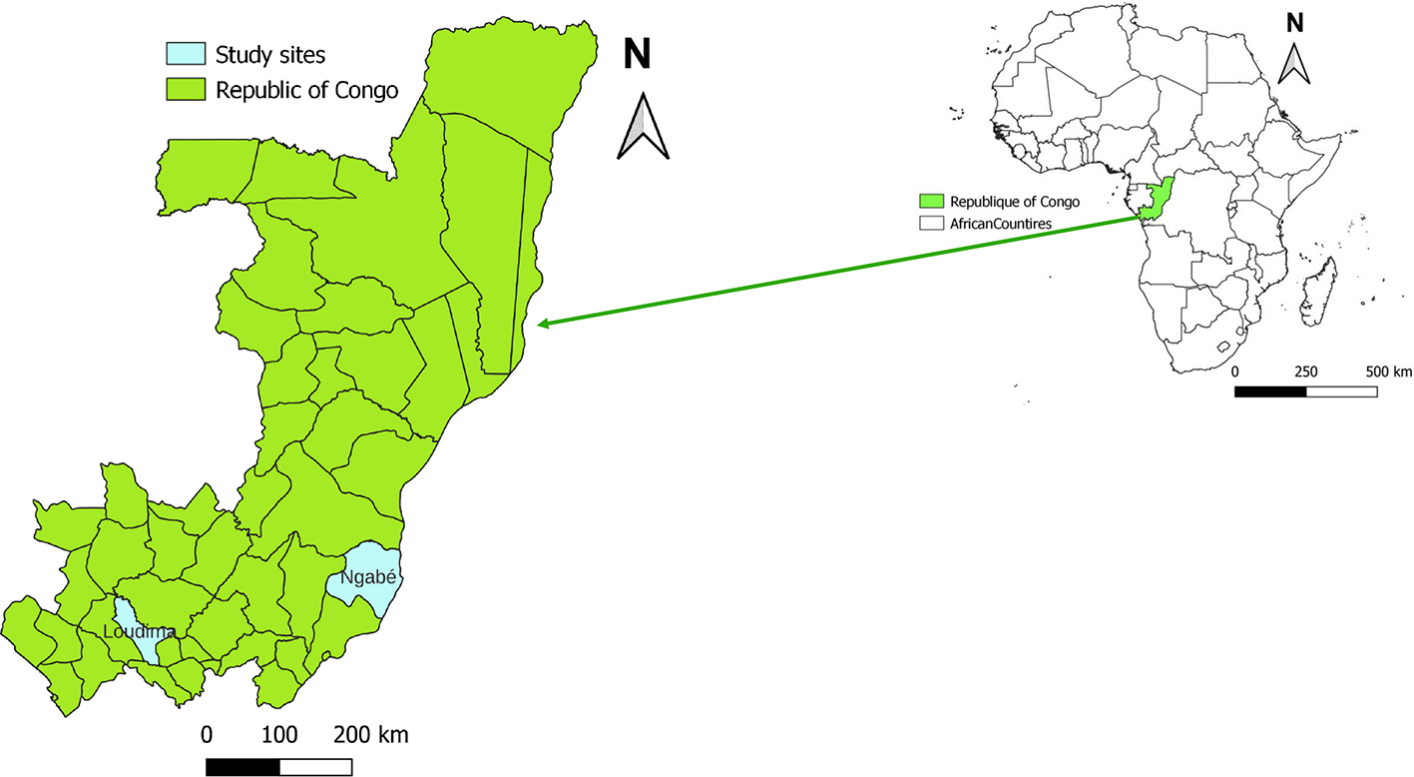
Map showing *Glossina* collection sites in Congo-Brazzaville. Red stars represent locations of surveyed villages.

The study was conducted in two villages of Ngabé district (Talangai and Ngobila) and two villages of Loudima district (Ditadi and Mont Belo), selected based on the distribution of HAT and because these sites remain active foci where the disease is still present [7]. These two sites are located in the southern region of the country (Fig. 1). Ngabé (3°12′52″S, 16°10′1″E) lies approximately 200 km north of the capital city, Brazzaville, within Pool Department. The Ngabé area includes several villages situated along the Congo River, characterized by a landscape of savannah interspersed with riparian forest galleries. Loudima (4°6′45″S, 13°3′30″E), located in Bouenza Department about 300 km south of Brazzaville, is predominantly covered by tall grasslands. The primary economic activity of the population in Loudima is subsistence agriculture, although livestock rearing is also practiced.

### Tsetse fly sampling and morphological identification

*Glossina* sampling was conducted in June and July 2021 using pyramid traps [30] deployed at various locations, including sites near rivers or swamps, shaded areas, and within villages. Traps were maintained in position for a minimum of three consecutive days in each village. A total of 51 traps were deployed: 34 in Loudima (17 in Ditadi and 17 in Mont Belo) and 17 in Ngabé (6 in Talangai and 11 in Ngobila). The geographical coordinates of each trap site were recorded using a GPS device (eTrex^®^ 10, Garmin Ltd., Olathe, KS, USA). Traps were spaced at least 100 meters apart and monitored twice daily, at 10:00 and 16:00. Detailed information on the sampling sites, including collection dates, trap numbers, and GPS coordinates, are provided in Supplementary Table S1. Captured *Glossina* specimens were morphologically identified to the species level by trained entomologists in the Republic of the Congo using a binocular magnifying glass and dichotomous identification keys [18]. *Glossina* samples were then preserved in microtubes containing 70% ethanol and were subsequently transferred to IHU-Méditerranée Infection laboratory (Marseille, France) for MALDI-TOF MS and molecular analyses. This transfer was authorized under import authorization number ER55-2021, permitting the import of animal-origin samples from non-European Union countries.

### *Glossina* dissection

*Glossina* specimens were rinsed with distilled water and dried on filter paper prior to dissection [31]. The two wings, three legs, and thoraxes of each specimen were dissected using a sterile surgical blade and individually placed in 1.5 mL Eppendorf tubes for MALDI-TOF MS analysis [14]. Each abdomen was longitudinally bisected; one half was stored at −20 °C as a backup sample, while the other half was used for DNA extraction and molecular analysis, including species or subspecies identification and screening for microorganisms.

### Genomic DNA extraction and molecular characterization of *Glossina*

Genomic DNA was extracted from half of the abdomen of each *Glossina* specimen following overnight incubation at 56 °C in a 1.5 mL microcentrifuge tube containing 180 μL of G2 lysis buffer and 20 μL of Proteinase K (QIAGEN, Hilden, Germany). DNA extraction was performed using an EZ1 DNA Tissue Kit (QIAGEN), according to the manufacturer’s instructions. The final eluate volume was 100 μL, and extracted DNA samples were stored at −20 °C until further analysis. Molecular characterization was performed on *Glossina* specimens whose MALDI-TOF MS spectra were used to construct the reference database, as well as on those exhibiting discrepancies between morphological and MALDI-TOF MS identification. Species and subspecies identification was conducted through PCR amplification and sequencing of three genetic markers: two mitochondrial cytochrome c oxidase subunit I regions, referred to in this study as COI-1 [28] and COI-2 [26], and one nuclear marker, the internal transcribed spacer 1 (ITS1) gene [26]. PCR products were visualized by electrophoresis on 1.5% agarose gels. All positive amplicons were purified and directly sequenced using a BigDye Terminator Cycle Sequencing Kit (Applied Biosystems, Foster City, CA, USA) on a 3500 Series Genetic Analyzer (Applied Biosystems). The resulting sequences were assembled and manually curated using ChromasPro software (version 1.7; Technelysium Pty Ltd., Tewantin, Australia) and subsequently compared to reference sequences available in the GenBank database (National Center for Biotechnology Information, NCBI) for species or subspecies identification. Sequences of *COI-1* and *COI-2* genes from selected specimens were used to construct phylogenetic trees. Sequence alignment was performed using BioEdit software with the ClustalW multiple alignment algorithm, and the aligned sequences were subsequently exported to MEGA X for phylogenetic analysis using the maximum likelihood method with 1,000 bootstrap replications.

### Sample preparation for MALDI-TOF analysis

Tubes containing the dissected body parts (i.e., legs, wings, and thorax) of each *Glossina* specimen were placed in a dry bath with open caps and incubated overnight at 37 °C to evaporate ethanol prior to sample homogenization [25]. Each body part was then homogenized in a defined volume of an extraction solution consisting of 70% formic acid and 50% acetonitrile (1:1, v/v; Fluka, Buchs, Switzerland). The extraction solution volumes were established at 50 μL for legs, 50 μL for wings, and 150 μL for thoraxes. Homogenization of samples was performed using a TissueLyser (QIAGEN) with a pinch of glass beads (Sigma, Lyon, France) as the mechanical disruptor. The homogenization process consisted of 2 cycles of 3 min for wings, 3 cycles for legs, and 4 cycles for thoraxes, all at a frequency of 30 Hz. The supernatant of each protein extract was spotted onto a polished steel MALDI-TOF MS target plate and overlaid with a matrix solution consisting of a saturated solution of α-cyano-4-hydroxycinnamic acid (HCCA), as previously described [25, 43]. Legs from a *Rhipicephalus sanguineus* s.s. tick colony, laboratory reared as previously described [36, 44], were spotted in duplicate on each MALDI-TOF target plate as a quality control for sample preparation and MS spectra acquisition. Additionally, the matrix solution alone was applied in duplicate as a negative control to monitor background signals and potential contamination.

### MALDI-TOF MS parameters and spectra analysis

Spectral profiles were acquired using a Microflex LT MALDI-TOF mass spectrometer (Bruker Daltonics GmbH, Bremen, Germany) operating in linear positive ion mode, with a laser frequency of 50 Hz and a mass range of 2–20 kDa. The accelerating voltage was set at 20 kV, with an extraction delay of 200 ns. Each spectrum was generated from 240 laser shots distributed across six regions of a single sample spot. Data acquisition was performed automatically using AutoXecute within FlexControl software, version 2.4 (Bruker Daltonics GmbH) [42]. Mass spectra were initially inspected visually using FlexAnalysis software, version 3.3 (Bruker Daltonics GmbH). The spectral data were then exported to ClinProTools, version 2.2 and MALDI-Biotyper, version 3.0 (Bruker Daltonics GmbH) for preprocessing, including smoothing, baseline subtraction, and peak picking. All MS spectra exhibiting low signal intensity (<3000 a.u.) and/or high background noise were deemed poor quality (non-compliant) and excluded from further analysis [13, 23, 31]. The reproducibility and specificity of *Glossina* MS spectra were assessed using cluster analysis (dendrogram) and principal component analysis (PCA). Main spectrum profiles (MSPs), generated from four replicate spots per sample, were used for clustering analysis via the MSP dendrogram function in MALDI-Biotyper, version 3.0 to evaluate spectral similarity across samples. Additionally, PCA was performed using ClinProTools, version 2.2 (default settings) to explore the distribution patterns of MS spectra obtained from different anatomical compartments (i.e., legs, wings, and thorax) of *Glossina* specimens. Cluster analysis (MSP dendrogram) was performed using MALDI-Biotyper, version 3.0, based on comparisons of MSPs generated from each sample. Samples were grouped according to the similarities in their protein mass spectra, considering both m/z values and peak intensities. Additionally, ClinProTools, version 2.2 was used to identify discriminatory peaks between the two *Glossina* species for each anatomical compartment. The most intense MS peaks per species and per body part were analyzed using ClinProTools, version 2.2 to evaluate their discriminatory power, as previously described [17]. Based on the peak lists generated for each species and compartment, the top 10 and top 20 most intense m/z peaks were selected for inclusion in a genetic algorithm (GA) model. These selected peaks yielded recognition capability (RC) values associated with the highest cross-validation (CV) scores. The presence or absence of all discriminating peak masses generated by the GA model was controlled by comparing the average spectra from each species per body part [23].

### Database creation and blind test for tsetse fly identification

The MALDI-TOF MS reference database was constructed using spectra from five specimens per body part (i.e., wings, legs, and thorax) and per species, using the MALDI-Biotyper software (Bruker Daltonics GmbH). MSPs were generated using an unbiased algorithm that integrates information on peak position, intensity, and frequency. All specimens included in our MS spectral database (DB) which already contained spectra from various arthropod species had been molecularly identified [10]. The remaining MS spectra were subjected to blind testing against this updated database. Identification confidence was assessed using the Log Score Value (LSV) provided by MALDI-Biotyper, which ranges from 0 to 3. The LSV reflects the degree of similarity between the test spectrum and the reference MSPs in the database, with higher scores indicating greater identification reliability.

### Detection of microorganisms

Quantitative PCR (qPCR) was used to detect microorganisms by targeting *Rickettsia* spp., *Coxiella burnetii*, bacteria of the Anaplasmataceae family, *Bartonella* spp., *Borrelia* spp. and *Trypanosoma* spp*.* with specific primers and probes, as previously described [31, 40]. The composition of the reaction mixture and the qPCR cycling conditions were identical to those previously described [40]. Amplifications were carried out on a CFX96 Real-Time system (Bio-Rad Laboratories, Foster City, CA, USA). For each qPCR assay, genomic DNA from *Rickettsia montanensis*, *Bartonella elizabethae*, *Anaplasma phagocytophilum*, *Coxiella burnetii*, *Borrelia crocidurae*, and *Trypanosoma congolense* was used as a positive control. DNA extracted from *Rhipicephalus sanguineus* s.s. ticks reared in our laboratory and confirmed to be free of the target microorganisms served as the negative control. Samples were considered positive when the cycle threshold (Ct) value was ≤36 for bacterial targets [31] and ≤38 for *Trypanosoma* spp. [52].

All *Trypanosoma* spp. qPCR-positive samples were further analyzed by conventional PCR to identify the species, using primers targeting the 28S large subunit (LSU) rRNA gene (F2: 5′–ACCAAGGAGTCAAACACACG–3′; R1: 5′–GACGCCACATATCCCTAAG–3′), which amplify a ~ 550 bp fragment [40]. PCR products displaying the expected band on agarose gel electrophoresis were purified and directly sequenced using a commercial Big Dye Terminator Cycle Sequencing Kit (Applied Biosystems, Perkin Elmer). The resulting sequences were edited with ChromasPro software and compared to reference *Trypanosoma* sequences available in the GenBank database for species identification. Primer details are provided in Supplementary Table S2.

### Evaluating the potential of MALDI-TOF MS to differentiate infected and uninfected *Glossina* specimens

To evaluate the potential of MALDI-TOF mass spectrometry for distinguishing *Glossina* specimens infected or uninfected with *Trypanosoma* spp. (as determined by molecular assays), MS spectra were compared according to infection status for each body part (legs, wings, and thorax) and for each *Glossina* species. PCA was first performed using ClinProTools, version 2.2 to explore unsupervised separation between infected and uninfected groups. In a second step, spectra were exported to ClinProTools for a peak-based comparison between infection statuses: mean spectra of each group were generated and a peak-list analysis was performed. Peaks were considered potentially discriminant if (i) the intensity fold change between infected and uninfected groups was greater than 2.0 or lower than 0.5, and if (ii) these last peaks were present in at least 30% of spectra in the dataset, to ensure sufficient representativeness. Peaks meeting these criteria were regarded as potential candidates for discriminating infected from uninfected specimens.

## Results

### Sampling, morphological identification, and molecular characterization of *Glossina*

A total of 265 *Glossina* specimens were collected from two localities: 200 from Loudima and 65 from Ngabé. Based on morphological identification using the taxonomic key of Brunhes et al. (1994), the specimens were classified into two groups: *Glossina palpalis* and *Glossina fuscipes*. All specimens from Loudima belonged to the *G. palpalis* group, whereas those from Ngabé were identified as members of the *G. fuscipes* group (Table 1).

**Table 1:** Overview of molecular characterization results for *Glossina* species and subspecies using *COI-1*, *COI-2*, and *ITS1* gene markers*.*

Morphological ID		*COI-1*			*COI-2*			*ITS1*		
	Number per group (*n*)	Molecular ID	Accession number	Proportion of coverage/identity (range)	Molecular ID	Accession number	Proportion of coverage/identity (range)	Molecular ID	Accession number	Proportion of coverage/identity (range)
*Glossina palpalis* group	10^a^	*G. palpalis*	EF531202	91–100% /97.07–97.80%	*G. p. palpalis*	MN750714	99–100%/99.47–100%	*G. p. palpalis*	LR813478	98–100%/97.69–98.98%
	48^b^	*G. palpalis*	EF531202	91–100% /97.07–97.80%	*G. p. palpalis*	MN750714	99–100%/99.47–100%	*G. p. palpalis*	LR813478	98–100%/97.69–98.98%
*Glossina fuscipes* group	9^a^	*G. f. fuscipes*	KP979583 KP979584	98–100%/99.22–99.86%	*G. f. quanzensis*	HQ387030	100%/100%	*G. f. quanzensis*	EU591941	100%/100%
	7^b^	*G. f. fuscipes*	KP979583 KP979584	98–100%/99.22–99.86%	*G. f. quanzensis*	HQ387030	100%/100%	*G. f. quanzensis*	EU591941	100%/100%

a Specimens used for the creation of the MALDI-TOF MS reference database.

b Specimens obtaining discrepant results between morphological and MALDI-TOF MS identification.

ID, Identification; *COI*, cytochrome oxidase; *ITS*, internal transcribed spacer.

To validate the morphological identifications, conventional PCR followed by sequencing were performed on ten specimens from the *G. palpalis* group and nine from the *G. fuscipes* group, targeting three genetic markers: *COI-1*, *COI-2*, and *ITS1*.

For specimens belonging to the *G. palpalis* group, BLAST analyses of *COI-1* gene sequences revealed coverages ranging from 91% to 100% and identities ranging from 97.07% to 97.80% with *Glossina palpalis* sequences available in GenBank (accession number EF531202) (Table 1). Moreover, BLAST analyses of *COI-2* and *ITS1* gene sequences showed coverages ranging from 98% to 100% and identities ranging from 97.69% to 100% to *G. palpalis palpalis* sequences available in GenBank (accession numbers MN750714 and LR813478) (Table 1).

For the specimens from the *Glossina fuscipes* group, BLAST analyses of the *COI-1* gene sequences revealed coverages ranging from 98% to 100% and identities ranging from 99.22% to 99.86% with *Glossina fuscipes fuscipes* sequences in GenBank (accession numbers KP979583, KP979584) (Table 1). Analyses of the *COI-2* and *ITS1* genes showed 100% coverage and 100% identity for both genes, matching *Glossina fuscipes quanzensis* sequences (accession numbers HQ387030 and EU591941) (Table 1). These molecular results support the conclusion that the analyzed *Glossina* specimens belong to the *G. palpalis* and *G. fuscipes* species groups, and more specifically to the subspecies *G. p. palpalis* and *G. f. quanzensis*.

**Supplementary Figure S1:**
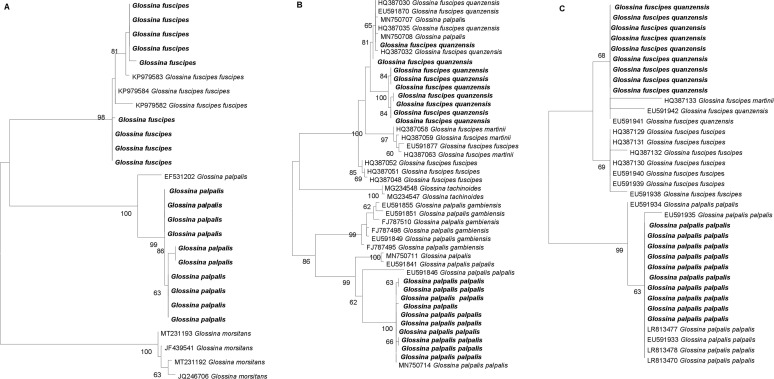
Maximum likelihood phylogenetic trees constructed using sequences from *Glossina* specimens whose MS spectra were included in the MALDI-TOF MS reference database. (A) Phylogenetic tree based on the *COI-1* gene. (B) Phylogenetic tree based on the *COI-2* gene. (C) Phylogenetic tree based on the *ITS1* gene.

*The COI-1*, *COI-2*, and *ITS1* sequences obtained from *Glossina* specimens of both subspecies were used to construct a phylogenetic tree (Supplementary Figure S1). These trees show that the sequences generated in this study consistently cluster with reference sequences of species within the *G. palpalis* and *G. fuscipes* groups available in GenBank for the *COI-1* gene (Supplementary Figure S1A), and with those of the subspecies *G. p. palpalis* and *G. f. quanzensis* for the *COI-2* and *ITS1* genes, respectively (Supplementary Figures S1B and S1C).

### MS spectra analysis: reproducibility and specificity across *Glossina* species and body parts

The legs, wings, and thorax of 265 *Glossina* specimens were subjected to MALDI-TOF MS analysis. Of the 795 spectra obtained (265 specimens × 3 body parts), 57 (7.2%) of them were excluded due to their non-conformity based on the quality criteria established previously [13, 23, 31]. Briefly, MS spectra with low signal intensity (<3000 a.u.) and/or high background noise were classified as non-compliant. Then, 11, 15, and 31 spectra, coming from wings, legs, and thoraxes, respectively, of both species, were excluded from the analysis. The proportion of compliant spectra varied per body part and species, with overall highest results for wings (95.8%), followed by legs (94.3%), and thoraxes (88.3%) (Table 2). More specifically, in *G. palpalis* specimens, the proportions of compliant spectra were 98.0% for wings, 96.5% for legs, and 93.5% for thoraxes. For wings, legs, and thoraxes of *G. fuscipes* specimens, the respective values were 89.2%, 87.7%, and 72.3% (Table 2).

**Table 2:** MALDI-TOF MS identification of *Glossina* species collected in the Republic of the Congo according to body part.

		Conform spectra (*n*)^#^			Concordant ID with morphological classification		
*Glossina* species		Wings	Legs	Thoraxes	Wings	Legs	Thoraxes
*G. p. palpalis*	Number of spectra	196 (191)	193 (188)	187 (182)	168	177	157
(*n* = 200)	Proportion (%)	98.0%	96.5%	93.5%	88.0%	94.1%	86.2%
	Range of LSVs				[1.70–2.83]	[1.76–2.65]	[1.72–2.71]
	Proportion (%)*				85.3%	92.6%	82.4%
*G. f. quanzensis*	Number of spectra	58 (53)	57 (52)	47 (42)	53	52	35
(*n* = 65)	Proportion (%)	89.2%	87.7%	72.3%	100%	100%	83.3%
	Range of LSVs				[1.81–2.44]	[1.95–2.48]	[1.78–2.52]
	Proportion (%)*				100%	100%	81.0%
Total		254 (95.8%)	250 (94.3%)	234 (88.3%)	221 (90.6%)	229 (95.4%)	192 (85.7%)

# Remaining MS spectra after upgrading home-made spectra database with five specimens per body part.

* Proportion of spectra concordantly identified with a relevant LSV (i.e., >1.8).

LSV, Log score value; ID, identification.

**Figure 2:**
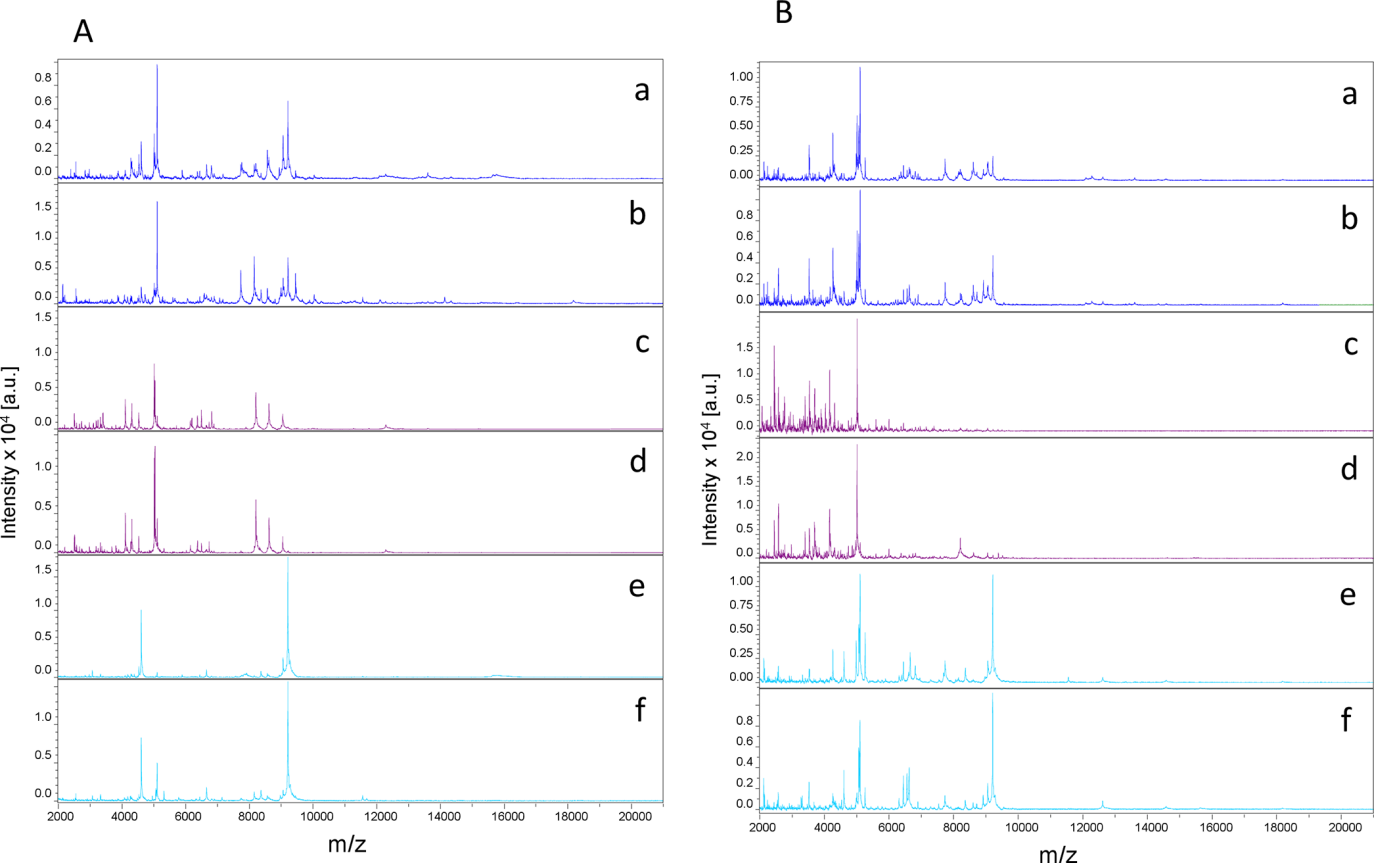
Comparison of representative MS spectra of two *Glossina* species using Flex analysis v.3.3. (A) Spectra of legs (a, b), thoraxes (c, d), and wings (e, f) of *Glossina fuscipes quanzensis*. (B) Spectra of legs (a, b), thoraxes (c, d), and wings (e, f) of *Glossina palpalis palpalis*; a.u., arbitrary units; m/z, mass-to-charge ratio.

**Figure 3:**
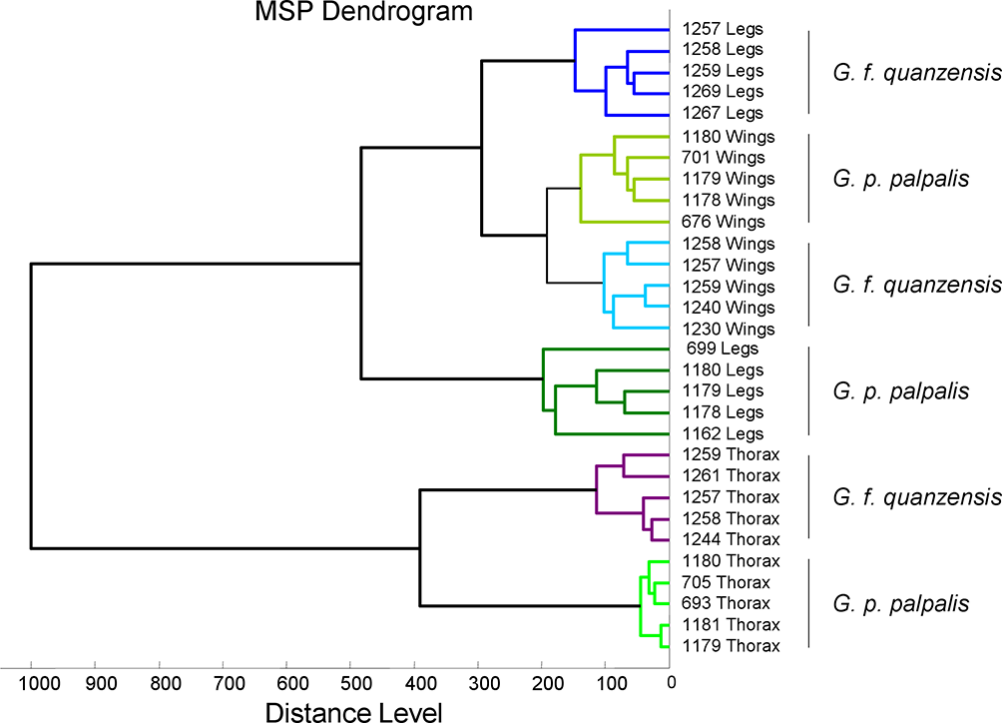
MSP dendrogram of MALDI-TOF mass spectra of *Glossina* legs, thoraxes, and wings, from specimens selected for the creation of the reference database created by MALDI-Biotyper, version 3.0. Distance units correspond to the relative similarity of the MS spectra. The same color code was used for the same body parts of the same *Glossina* species. a.u., arbitrary units; G.f., *Glossina fuscipes*; G.p., *Glossina palpalis*; MSP, main spectrum profile; m/z, mass-to-charge ratio.

Visual comparison of compliant MS spectra revealed intra-species reproducibility across body parts, as well as inter-species specificity according to body region (Fig. 2). In addition, spectral differences were observed between body parts within the same species. To further confirm the reproducibility and specificity of MS spectra based on *Glossina* species and body parts, a clustering analysis was performed. For this analysis, five representative spectra per body part and per species were selected. The resulting MSP dendrogram showed that specimens of the same species clustered together according to body part, highlighting both the reproducibility and the specificity of protein profiles for each anatomical region within each *Glossina* species (Fig. 3). It is noteworthy that thorax MS profiles of both *Glossina* species clustered in another branch of the dendrogram, highlighting a more pronounced difference in profile for this compartment (Fig. 3).

### MALDI-TOF MS database construction and blind validation for *Glossina* subspecies identification

The 10 specimens of *G. p. palpalis* and 9 specimens of *G. f. quanzensis*, for which identification was confirmed molecularly, were used for the creation of reference MS spectra. Then, among them, a total of 30 MS spectra, five per body part (legs, wings, and thorax) of each *Glossina* subspecies were selected for the construction of the reference MS spectra database. These MS spectra are accessible at the following link: https://doi.org/10.35081/kzhz-jt49. The MS spectra of the remaining specimens (i.e., those not included in the database) were queried against the reference MS DB, per body part for each species (Table 2).

**Figure 4:**
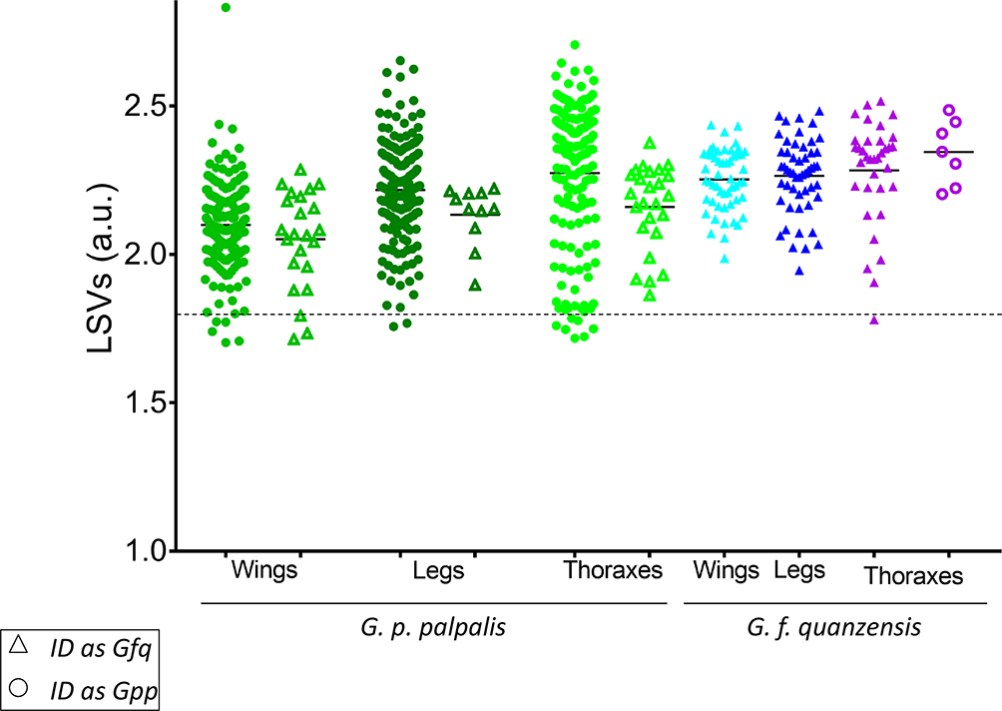
Comparison of LSVs from MS spectra of the wings, legs, and thoraxes of the two *Glossina* subspecies obtained through query against the homemade MS reference database. Triangles represent MS spectra identified as *G. f. quanzensis*, and circles represent those identified as *G. p. palpalis*. Solid circles and solid triangles represent MS spectra with identifications consistent with morphological identification of *Glossina* subspecies. Empty circles and empty triangles indicate spectra for which identifications were cross-referenced with morphological identification. The dotted line indicates the threshold for reliable identification (LSV > 1.8). Abbreviations: LSV, logarithmic score value; a.u., arbitrary units.

For *G. p. palpalis* and for *G. f. quanzensis* specimens, 89.5% (*n* = 502/561) and 95.2% (*n* = 140/147), respectively of the spectra from the three body parts corroborated the morphological identification. However, as LSVs should reach a threshold value (i.e., ≥1.8) to consider arthropod species identification reliable [14, 42], here, the proportions of relevant identification were 87.0% (*n* = 488/561) for *G. p. palpalis* and 94.6% (*n* = 139/147) for *G. f. quanzensis* (Table 2, Fig. 4). Interestingly, the best proportion of identification independently of the species was obtained for legs with 94.6% (*n* = 227/240), followed by wings (88.5%, *n* = 216/244), and thorax (82.1%, *n* = 184/224). Overall, 90.7% (*n* = 642/708) and 88.6% (*n* = 627/708) of the MS spectra queried against the DB were identified, respectively concordantly or concordantly and relevantly with morphological classification.

**Supplementary Figure S2:**
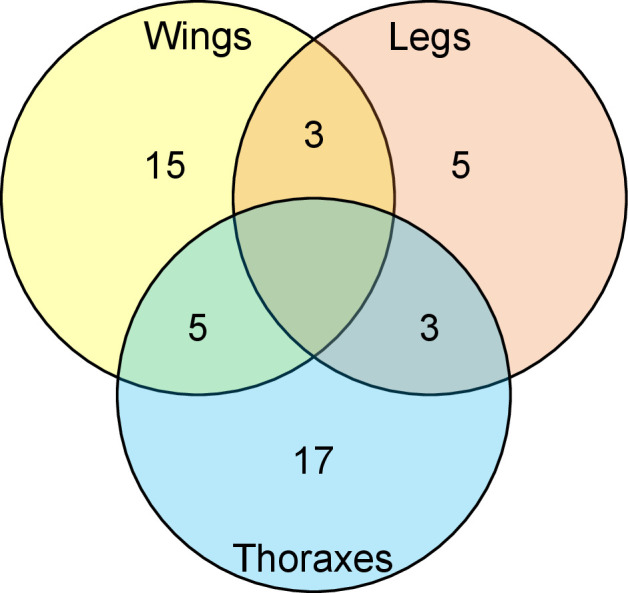
Classification of the 48 *G. p. palpalis* specimens for which discrepant identifications were obtained with MS spectra for at least one body part. The number of MS spectra cross-identified as *G. f. quanzensis* per body part is represented in the Venn diagram. The groups that were compared are indicated in each circle. The number of MS associated with each category are indicated.

Concerning the 66 out of 708 (9.3%) MS spectra classified as compliant but for which discrepancy occurred between MS and morphological identification, a cross-*Glossina* species identification was obtained (Fig. 4). The vast majority of these cross-identifications (95.5%, *n* = 63/66) reached the threshold (LSVs ≥ 1.8). For *G. f. quanzensis*, cross-identifications were exclusively observed in thoracic spectra (*n* = 7). Considering the results of the three body parts, relevant and concordant identification were obtained for the other two body parts (i.e., wings and legs), confirming the morphological classification as *G. f. quanzensis*. For *G. p. palpalis*, cross-identifications were obtained for legs (*n* = 11), wings (*n* = 23), and thorax (*n* = 25) MS spectra (Fig. 4). If the results of the three body parts were taken into account, the results of morphological and MS classification for 37 and 11 specimens were concordant for two body parts as *G. p. palpalis* and *G. f. quanzensis*, respectively (Supplementary Figure S2). Finally, overall, the proportion of discrepancy between morphological and MS identification was 4.4% (*n* = 11/250), if the MS identification of the three body parts per specimens was considered (Supplementary Figure S2).

### Biomarkers on legs, wings, and thorax distinguishing two *Glossina* species

To identify discriminating MS peaks among the wings, legs, and thorax of the two *Glossina* subspecies, MS spectra from specimens whose MALDI-TOF MS identification was concordant with morphological identification were analyzed. Specifically, 168, 177, and 157 MS spectra from the wings, legs, and thorax of *G. p. palpalis*, as well as 53, 52, and 35 MS spectra from the wings, legs, and thorax of *G. f. quanzensis*, were analyzed using the GA tool in ClinProTools. Selection of the 10 and 20 most intense mass peaks per body part and per species, based on the average spectrum peak reports, resulted in 31 and 56 distinct MS peaks, respectively (Supplementary Table S3). Incorporating these peak lists into the GA model yielded recognition capability (RC) and cross-validation (CV) values of 97.3% and 92.1% for the top ten peaks, and 98.3% and 94.8% for the top twenty peaks. These results highlight that the top ten and top twenty mass peak lists per species represent the most informative MS features for distinguishing the two *Glossina* species, regardless of the body part analyzed. Comparison of the top twenty peak lists across the three body parts for both *Glossina* species revealed that only six mass-to-charge (m/z) values (2070.7, 2199.6, 2573.7, 4174.8, 5064.3, and 5105.9) were shared, further supporting the high anatomical and species specificity of the MS spectra (Supplementary Table S3).

### Molecular identification of tsetse flies revealing discrepancies between morphological and MALDI-TOF MS identifications

*Glossina* specimens exhibiting discrepancies between morphological and MS identification were further analyzed using molecular identification with three genetic markers (*COI-1*, *COI-2*, and *ITS1*) to resolve their classification. This subset included 48 individuals of *G. p. palpalis* and seven of *G. f. quanzensis*, for which the MS spectra from at least one body part exhibited a discrepancy. The results of the BLAST analysis of the sequences from the three genes (*COI-1*, *COI-2*, and *ITS1*) confirmed the morphological identification. Specifically, specimens of *G. p. palpalis* misidentified by MALDI-TOF MS as *G. f. quanzensis* were correctly identified by molecular analysis as *G. p. palpalis*, and conversely, specimens of *G. f. quanzensis* misidentified by MALDI-TOF MS as *G. p. palpalis* were confirmed as *G. f. quanzensis* by molecular analysis. Sequence coverage ranged from 91% to 100%, with identity levels between 96.47% and 100% compared to the reference sequences of *G. p. palpalis* and *G. f. quanzensis* available in GenBank (Table 1). Sequences from 15 specimens of *G. p. palpalis* and 15 specimens of *G. f. quanzensis* obtained from the *COI-1*, *COI-2,* and *ITS1* genes were deposited in the GenBank database under the following accession numbers: PQ725737–PQ725766 for *COI-1*, PQ676200–PQ676229 for *COI-2*, and PQ730106–PQ730135 for *ITS1*. The deposited sequences include those from individuals whose MS spectra were used to construct the MALDI-TOF MS reference database.

### Detection of microorganisms

In terms of microorganisms, *Trypanosoma* spp. DNA was detected in 30 specimens (11.3%; *n* = 30/265) of *Glossina*, including 25 specimens of *G. p. palpalis* and five specimens of *G. f. quanzensis*. Amplification and sequencing yielded 22 usable sequences, of which 19 originated from *G. p. palpalis* specimens and three from *G. f. quanzensis* specimens. BLAST analysis of these 22 sequences showed 100% coverage and identities ranging from 98.12% to 99.20% with the reference sequence of *Trypanosoma congolense* riverine/forest type (GenBank accession number: U22319). Due to the high similarity among the *T. congolense* sequences obtained, three sequences from *G. p. palpalis* specimens and three from *G. f. quanzensis* specimens were selected and deposited in the GenBank database under accession numbers PV061377–PV061382. None of the *Glossina* specimens were positive for *Rickettsia* spp., *Coxiella burnetii*, bacteria of the family *Anaplasmataceae*, *Bartonella* spp., and *Borrelia* spp.

### Assessment of MALDI-TOF MS potential for discriminating infected and uninfected *Glossina* specimens

**Supplementary Figure S3:**
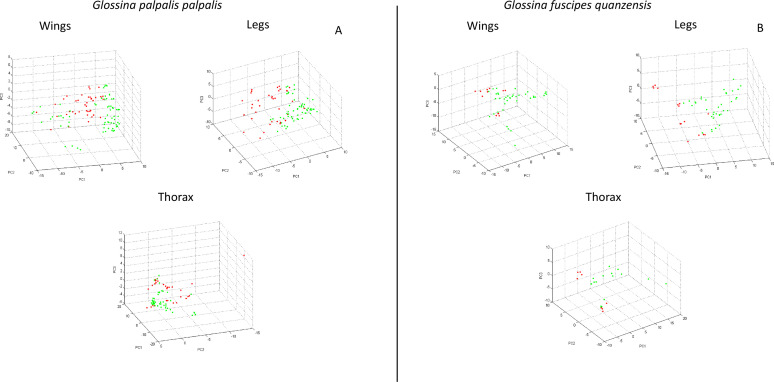
Principal component analysis (PCA) based on MS spectra from the legs, wings, and thoraxes of *G. p. palpalis* (A) and *G. f. quanzensis* (B), either infected or uninfected with *T. congolense*. Red dots represent MS spectra from *Glossina* infected with *T. congolense*, whereas green dots correspond to uninfected specimens.

The comparison of MALDI-TOF MS spectra from *G. p. palpalis* and *G. f. quanzensis* specimens, either infected or uninfected with *Trypanosoma*, did not reveal a clear distinction between the two groups. PCA performed on spectra from different body parts (wings, legs, and thorax) showed substantial overlap between infected and uninfected specimens, regardless of the species analyzed (Supplementary Figure S3). A slight tendency for clustering of thoracic spectra was observed in *G. p. palpalis*, but without clear separation. In *G. f. quanzensis*, no significant distinction could be detected in any compartment (Supplementary Figure S3). Consistently, peak-based analysis in ClinProTools of paired comparisons per species, body part and infectious status, revealed that none of the MS peaks exhibiting variations above 2.0 or below 0.5, were found in at least on third of the dataset analyzed. These results indicated that infection-related to spectral differences, if they exist, were too far below the threshold to be perceived in the described experimental conditions.

## Discussion

MALDI-TOF MS is a cutting-edge technology that has revolutionized microbiological diagnostics by enabling the rapid and accurate identification of bacteria, parasites, viruses, and archaea [19, 51]. In the field of medical entomology, MALDI-TOF MS has emerged as a valuable tool for arthropod identification, offering a rapid, reliable, and high-throughput alternative to conventional morphological and molecular methods [63]. The technique has demonstrated efficacy in distinguishing various vector species, including mosquitoes [23, 27], ticks [25, 44], bedbugs [9, 45], and other arthropods of medical importance [51, 63]. Currently, MALDI-TOF MS is recognized as a reliable, rapid, and cost-effective method especially in terms of reagent use for the identification of arthropods [51, 63].

Despite its advantages, several limitations hinder the widespread adoption of MALDI-TOF MS in large-scale medical entomology projects, particularly in African countries [1]. These include the high initial investment cost of the instrument (approximately €200,000), annual maintenance fees of around 10% of the purchase cost, and the absence of universally accessible reference MS spectra databases equivalent to GenBank [22, 51]. Although the scientific community has increasingly promoted the sharing of reference spectra leading to the publication of databases for certain arthropod families over the past decade [22, 51, 63], this practice has yet to be standardized or widely implemented.

Based on morphological observations, the *Glossina* specimens examined in this study were identified as belonging to the *G. palpalis* and *G. fuscipes* species groups. Due to the difficulty or, in some cases, impossibility of reliably distinguishing subspecies within these groups using morphological criteria alone [26], identification was restricted to the species level. The *Glossina palpalis* group comprises two subspecies: *G. p. gambiensis* and *G. p. palpalis*, which exhibit subtle morphological differences such as body size, coloration, the shape and size of male genitalia, and the pattern of setae on the tergite that make them difficult to distinguish without specialized tools, particularly when dealing with field-collected or damaged specimens [26]. Also, *G. p. gambiensis* is primarily distributed in humid savannah regions, whereas *G. p. palpalis* is predominantly found in forested areas [49]. The *G. fuscipes* group comprises three main subspecies *G. f. fuscipes*, *G. f. martinii*, and *G. f. quanzensis* which are morphologically very similar and therefore difficult to distinguish using classical taxonomic methods [26]. According to previous studies, one species from the *G. palpalis* group (*G. p. palpalis*) and two from the *G. fuscipes* group (*G. f. fuscipes* and *G. f. quanzensis*) are present in the Republic of the Congo, each with clearly distinct geographical distributions [6, 7].

In this study, the *COI-1*, *COI-2*, and *ITS1* genes [26, 28] were used not only to validate the morphological identification of the two species groups (*G. palpalis* and *G. fuscipes*), but also to overcome the limitations of morphological methods by enabling subspecies-level identification within each group. The ultimate goal was to include spectra from confidently identified specimens in the MALDI-TOF MS reference database. The results showed that the *COI-1* gene is effective in discriminating between species groups, but insufficient for reliably distinguishing subspecies within each group. This limitation is largely due to the absence of reference sequences of the *COI-1* gene for certain subspecies such as *G. p. palpalis*, *G. p. gambiensis*, and *G. f. quanzensis* in the GenBank database. However, the *COI-2* and *ITS1* sequences allowed for the identification of two subspecies, *G. p. palpalis* and *G. f. quanzensis*. These findings underscore the importance of using multiple complementary genetic markers to achieve robust and reliable molecular identification. The absence or limited availability of gene sequences for certain *Glossina* subspecies highlights the need to enrich the GenBank database with sequences from diverse geographical regions. Such efforts are essential to capture the genetic diversity required for accurate subspecies discrimination.

The results of our molecular identification of *Glossina* specimens (*G. p. palpalis* and *G. f. quanzensis*) are consistent with previous studies conducted in the Republic of the Congo, which reported the presence of these subspecies [38]. In this country, these two *Glossina* subspecies exhibit distinct geographical distributions: *G. p. palpalis* is found in the southern part of the country, extending southward to Brazzaville, particularly around the Djoué River, whereas *G. f. quanzensis* ranges from Gamboma to the Ngabé corridor along the banks of the Congo River [38]. Despite the existence of clearly defined distribution areas, a contact zone between the two subspecies has been observed near Brazzaville, suggesting dynamic expansion of *G. p. palpalis* into the range of *G. f. quanzensis*, potentially displacing the latter [38]. This pattern is consistent with our morphological and molecular identification results, as each subspecies was predominantly found within its respective historical range [38]. Both *G. p. palpalis* and *G. f. quanzensis* are recognized as major vectors of HAT in West and Central Africa, including the Republic of the Congo [11, 37, 56].

In this study, MALDI-TOF MS was employed to differentiate between two *Glossina* subspecies based on mass spectra generated from the wings, legs, and thorax. Analysis of the MS spectra using visual inspection and MSP dendrograms revealed spectral specificity both at the subspecies level of *Glossina* and according to the body part analyzed. This resulted in the clustering of MS profiles by subspecies and by the three distinct body parts. Similar body part- and species-specific spectral patterns have previously been documented in other arthropods [14, 17, 23, 59]. To minimize subspecies misidentification, spectra from legs, wings, and thoraxes were independently queried in the database in separate blind analyses. This strategy of analyzing multiple body parts per specimen enhanced identification accuracy and confidence, while minimizing the risk of misidentification [4, 14, 23]. The proportion of high-quality MS spectra varied depending on the *Glossina* body part analyzed, with the highest quality observed for wings, followed by legs and thorax. Previous studies have demonstrated that multiple factors can influence MS spectral quality, including sample homogenization methods, the volume and composition of the extraction buffer, the engorgement status of the arthropod, storage conditions, and even the geographical origin of the specimen [41, 51].

Blind tests independently comparing spectra from the three body parts revealed that the proportion of concordant identifications between morphological and MALDI-TOF MS-based identification varied by body part, being highest for legs (95.4%; *n* = 229/240), followed by wings (90.6%; *n* = 221/244), and thorax (85.7%; *n* = 192/224). Similarly, the proportion of identifications considered reliable (LSV ≥ 1.8) [14, 42] was also higher for legs and wings than for thoraxes. The results of this study suggest that the wings and legs are the most suitable *Glossina* body parts for MALDI-TOF MS analysis, as they yielded higher proportions of high-quality MS spectra and correct identifications compared to thoraxes.

Interestingly, no specimens exhibited discordant identifications across all three body parts. This observation is consistent with findings in mosquitoes, where species identification can be achieved using the legs, thorax, or head, although the most reliable results are typically obtained from the thorax, followed by the legs [4].

Cross-identifications were observed among the MS spectra of the wings (*n* = 23), legs (*n* = 11), and thoraxes (*n* = 25) of *G. p. palpalis* specimens, whereas for *G. f. quanzensis*, cross-identifications were observed exclusively in spectra of the thoraxes (*n* = 7). Assuming that MALDI-TOF MS-based identification is considered valid when the MS spectra from at least two of the three body parts of a given individual are concordant with LSVs ≥ 1.8 according to past studies, cross-identification was observed in 11 *G. p. palpalis* specimens, representing fewer than 5%. Among these 11 specimens, three involved the leg–wing pair, three the leg–thorax pair, and five the wing–thorax pair.

However, the number of cross-identifications was corrected and/or minimized when the MALDI-TOF MS identification results from the three body parts (wings, legs, and thorax) of the *Glossina*, analyzed independently, were combined, as previously reported [14, 15, 17, 23, 59]. These findings reinforce previous studies [14, 15, 17, 23, 59] demonstrating that the accuracy and reliability of MALDI-TOF MS identification are improved when multiple body parts of arthropods are analyzed separately. This is notably the case for mosquitoes, with independent analyses of legs and thorax [17, 23, 59], as well as for ticks, with analyses of legs, capitulum, and half of the idiosoma [14].

For the 11 specimens showing cross-identification in the MS spectra of two body parts, BLAST analyses of the *COI-1*, *COI-2*, and *ITS1* gene sequences supported the morphological identification. Despite these cross-identifications, the overall correct identification rate remained around 95%, highlighting the robustness of the MALDI-TOF MS method for discriminating *Glossina* subspecies, as previously reported [33, 34]. Our results support the concept of integrative taxonomy, which advocates the use of multiple complementary techniques to achieve more accurate identification of arthropod species. This integrative approach is particularly valuable for distinguishing species complexes or closely related species groups, as previously proposed [48].

Selection of the ten or twenty most intense peaks from the wing, leg, and thorax spectra of the two *Glossina* subspecies resulted in high recognition (RC) and cross-validation (CV) scores 97.3% and 92.1% for the ten-peak model, and 98.3% and 94.8% for the twenty-peak model. These results demonstrate the ability of MALDI-TOF MS to discriminate *Glossina* subspecies and body parts based on spectral profiles, consistent with findings previously reported [17, 23]. However, the 10 most intense peaks are sufficient to achieve strong discrimination between *Glossina* subspecies, as the MS spectra from different body parts share few common peaks between subspecies, which likely explains the high rates of correct classification.

In terms of microorganisms, only *Trypanosoma* DNA closely related to *T. congolense* of the riverine/forest type was detected in the *Glossina* specimens analyzed at two study sites. Of note, *T. congolense* is the causative agent of AAT in cattle throughout much of sub-Saharan Africa. This disease has significant economic and public health implications due to its wide geographical distribution and the broad range of vertebrate hosts it can infect [3]. AAT manifests with symptoms such as fever, anemia, weight loss, and reduced milk production, leading to substantial economic losses for livestock farmers [24]. However, several studies have reported the presence of not only *T. congolense* but also other trypanosome species in *G. p. palpalis* and/or *G. f. quanzensis* in both the Republic of the Congo and Cameroon [7, 53, 54]. *Trypanosoma congolense* DNA has also been detected in other *Glossina* species, such as *Glossina pallidipes*, *Glossina brevipalpis*, and *Glossina austeni*, in other African countries [29, 46]. None of the *Glossina* specimens tested positive for any of the bacterial screened, including *Rickettsia* spp., *C. burnetii*, members of the *Anaplasmataceae* family, *Bartonella* spp., and *Borrelia* spp. The presence of DNA from the bacterial groups screened in this study has rarely been reported in the literature, except for *Wolbachia*, a widespread endosymbiont from the *Anaplasmataceae* family that plays a fundamental role in insect physiology, including metabolic regulation, fertility, and immune function [61]. Although some studies have reported the presence of a *Rickettsia* species phylogenetically related to *Rickettsia felis* in *Glossina morsitans submorsitans* in Senegal [39] and *Ehrlichia ruminantium* in *Glossina pallidipes* in Ethiopia [35], the role of these *Glossina* species in the transmission of these pathogens remains unproven*.*

Here, the comparison of MS spectra from different body parts of *G. p. palpalis* and *G. f. quanzensis* specimens, either infected or not with *T. congolense*, did not reveal any clear differences between the two groups. This absence of consistent spectral differences across all analyzed body parts may partly reflect the tissue tropism of *Trypanosoma*, which primarily develops in the digestive tract and, for *T. congolense*, also in the proboscis and salivary glands [2]. Consequently, legs and wings may harbor few or no parasites, thereby limiting infection-related proteomic changes detectable by MALDI-TOF MS in these tissues. Similar limitations of MALDI-TOF MS in discriminating between infected and uninfected arthropods have been reported in field-collected ticks, both hard and soft [37, 43, 63]. In contrast, several laboratory-based studies using artificially infected arthropods have demonstrated successful discrimination between infected and uninfected specimens [22, 23, 44, 61].

## Conclusion

MALDI-TOF MS has proven to be a reliable tool for the identification of *Glossina* subspecies. However, its application across a broader diversity of species and subspecies is essential to validate these findings and to develop a more comprehensive reference database. Our results support the use of legs and wings as preferred body parts for analysis, as these tissues consistently yield high-quality, reproducible, and specific spectra suitable for subspecies discrimination. Given the limitations inherent to each identification method, combining results from multiple techniques within an integrative taxonomy framework will enhance the accuracy of species and subspecies identification within the *Glossina* genus.

**List of abbreviations**
AAT: Animal African trypanosomiasis;
BLAST: Basic local alignment search tool;
*COI-1*
: Cytochrome c oxidase subunit 1;
*COI-2*
: Cytochrome c oxidase subunit 2;
Ct: Cycle threshold;
CV: Cross-validation;
GA: Genetic algorithm;
HAT: Human African trypanosomiasis;
*ITS1*
: Internal transcribed spacer I;
LSU: Large subunit;
LSV: Log score value;
MALDI-TOF MS: Matrix-assisted laser desorption/ionization time-of-flight mass spectrometry;
PCR: Polymerase chain reaction;
RC: Recognition capacity

## Data Availability

The MS reference spectra added to the MS database are freely available and can be downloaded from the following link: https://doi.org/10.35081/kzhz-jt49. All additional information from this study is presented in Supplementary Figures S1, S2, and S3, and Supplementary Tables S1, S2, and S3.

## Supplementary material

*Supplementary Table S1*: Location and distribution of pyramid traps used for tsetse fly sampling in June–July 2021. *Supplementary Table S2*: Primers and probes used for the identification of *Glossina* species and microorganism detection. *Supplementary Table S3*: Top twenty mass peak list per *Glossina* species using wings, thoraxes, and legs. The supplementary material of this article is available at https://www.parasite-journal.org/10.1051/parasite/2026007/olm.
