# Supplementary material for: Glossina from the Republic of the Congo: species identification by MALDI-TOF MS and research of associated micro-organisms
Source: Parasite. 2026 Feb 5;33:5. doi: 10.1051/parasite/2026007 (PMC12875062; doi:10.1051/parasite/2026007)
Supplement: Supplementary file 1 — Supplementary Table S1: Location and distribution of pyramid traps used for tsetse fly sampling in June–July 2021. [file parasite-33-5-s1.pdf]

Supplementary Table S1. Location and distribution of pyramid traps used for tsetse fly sampling in June–July 2021.

				GPS coordinates	
**Foci**	**Villages**	**Date**	**Trap number**	**Latitude**	**Longitude**
Loudima	Ditadi	06.16.2021	P1	-4,17768	13,007283
		06.16.2021	P2	-4,177096	13,0098
		06.16.2021	P3	-4,140769	12,911024
		06.16.2021	P4	-4,140348	12,910892
		06.16.2021	P5	-4,138226	12,911534
		06.16.2021	P6	-4,138708	12,912397
		06.16.2021	P7	-4,140784	12,912057
		06.16.2021	P8	-4,141195	12,912686
		06.16.2021	P9	-4,141087	12,913546
		06.16.2021	P10	-4,141584	12,913945
		06.16.2021	P11	-4,142235	12,914559
		06.16.2021	P12	-4,141396	12,916655
		06.16.2021	P13	-4,142257	12,91795
		06.16.2021	P14	-4,142892	12,917891
		06.16.2021	P15	-4,143082	12,919078
		06.16.2021	P16	-4,144331	12,917692
		06.16.2021	P17	-4,1451	12,915564
	Mont Belo	06.20.2021	P18	-4,146059	12,915459
		06.20.2021	P19	-4,145055	12,916999
		06.20.2021	P20	-4,174704	13,004211
		06.20.2021	P21	-4,174078	13,003444
		06.20.2021	P22	-4,175149	13,002093
		06.20.2021	P23	-4,1741	13,002793
		06.20.2021	P24	-4,177648	13,003749
		06.20.2021	P25	-4,181473	13,005941
		06.20.2021	P26	-4,180475	13,00682
		06.20.2021	P27	-4,178325	13,00637
		06.20.2021	P28	-4,17811	13,006199
		06.20.2021	P29	-4,175012	13,005768

		06.20.2021	P30	-4,175374	13,005312
		06.20.2021	P31	-4,176571	13,005642
		06.20.2021	P32	-4,177045	13,005741
		06.20.2021	P33	-4,181256	13,006193
		06.20.2021	P34	-4,194705	12,988253
Ngabé	Talangai	06.28.2021	P1	-3,279211	16,183818
		06.28.2021	P2	-3,279209	16,18381
		06.28.2021	P3	-3,279901	16,183524
		06.28.2021	P4	-3,277857	16,182965
		06.28.2021	P5	-3,277563	16,182587
		06.28.2021	P6	-3,277351	16,183291
	Ngobila	06.28.2021	P7	-3,277227	16,183547
		06.28.2021	P8	-3,285899	16,197161
		06.28.2021	P9	-3,285351	16,197573
		06.28.2021	P10	-3,28573	16,200444
		06.28.2021	P11	-3,288178	16,204185
		06.28.2021	P12	-3,288214	16,204674
		06.28.2021	P13	-3,289197	16,205773
		06.28.2021	P14	-3,28924	16,206286
		06.28.2021	P15	-3,288611	16,206016
		06.28.2021	P16	-3,291516	16,206726
		06.28.2021	P17	-3,291986	16,205981
