# Supplementary material for: Glossina from the Republic of the Congo: species identification by MALDI-TOF MS and research of associated micro-organisms
Source: Parasite. 2026 Feb 5;33:5. doi: 10.1051/parasite/2026007 (PMC12875062; doi:10.1051/parasite/2026007)
Supplement: Supplementary file 2 — Supplementary Table S2: Primers and probes used for the identification of Glossina species and microorganism detection. [file parasite-33-5-s2.pdf]

Supplementary Table S2. Primers and probes used for the identification of *Glossina* species and microorganism detection

**Targeted organism**	**Targeted gene**	**Primers (5'-3') and probes used for qPCR screening or sequencing**	**Refs**
Rickettsia spp.	gltA (RKND03)	f_GTGAATGAAAGATTACACTATTTAT r_GTATCTTAGCAATCATTCTAATAGC p_6FAM-CTATTATGCTTGCGGCTGTCGGTTC	[31]
Borrelia spp.	Bor (ITS4)	f_GGCTTCGGGTCTACCACATCTA r_CCGGGAGGGGAGTGAAATAG p_6FAM-TGCAAAAGGCACGCCATCACC	[31]
Anaplasmataceae	23S rRNA	f_TGACAGCGTACCTTTTGCAT r_GTAACAGGTTCGGTCCTCCA p_6FAM-GGATTAGACCCGAAACCAAG	[31]
Bartonella spp.	Barto (ITS2)	f_GATGCCGGGGAAGGTTTTC r_GCCTGGGAGGACTTGAACCT p_GCGCGCGCTTGATAAGCGTG	[31]
Coxiella burnetii	IS30A	f_CGCTGACCTACAGAAATATGTCC r_GGGGTAAGTAAATAATACCTTCTGG p_CATGAAGCGATTTATCAATACGTGTATG	[31]
Trypanosoma spp.	28S	f_AGATCTTGGTTGGCGTAG r_ATAACGTTGTGCTCAGTTTCC p_FAM- GGGAAGGATTTCGTGCCAACG	[40]
Glossina spp.	*COI**	f_GGTCAACAAATCATAAGATATTGG r_TAAACTTCAGGGTGACCAAAAAATCA	[28]
	*COI-2**	f_TTGATTTTTTGGTCATCCAGAAG r_TGAAGCTTAAATTCATTGCACTAATC	[26]
	*ITS-1**	f_GTGATCCACCGCTTAGAGTGA r_GCAAAAGTTGACCGAACTTGA	[26]
Trypanosoma spp.	28S *LSU**	f2_ACCAAGGAGTCAAACAGACG r1_GACGCCACATATCCCTAAG	[40]

*Primers used for conventional PCR; *COI*, cytochrome oxidase subunit; *LSU*: Large subunit; f, forward; r, reverse; p, probes.
