# Supplementary material for: Glossina from the Republic of the Congo: species identification by MALDI-TOF MS and research of associated micro-organisms
Source: Parasite. 2026 Feb 5;33:5. doi: 10.1051/parasite/2026007 (PMC12875062; doi:10.1051/parasite/2026007)
Supplement: Supplementary file 3 — Supplementary Table S3: Top twenty mass peak list per Glossina species using wings, thoraxes, and legs. [file parasite-33-5-s3.pdf]

Supplementary Table S3. Top twenty mass peak list per *Glossina* species using wings, thoraxes and legs.

		**Average peak intensity 10^3(a.u.)***					
		Wings		***Legs***		***Thoraxes***	
**MS peak number§**	**m/z (Da)**	***G. p. palpalis***	***G. f. quanzensis***	***G. p. palpalis***	***G. f. quanzensis***	***G. p. palpalis***	***G. f. quanzensis***
1	2070.7#	*5.54*	*5.76*	6.81	5.11	6.09	*7.88*
**2**	2124.9	***11.74***	2.59	***12.73***	2.24		
3	2138.9	3.26	*7.63*				
**4**	2199.6#	*6.68*	5.26	***8.85***	***9.25***	*8.85*	***21.94***
**5**	2233.8	*7.79*	4.71	***11.16***	6.26		
**6**	2443.6			*7.11*	3.04	***17.36***	6.33
7	2460.4					*9.17*	3.12
8	2550.2	5.09	*5.62*	5.9	*7.41*		
**9**	2573.7#	*6.79*	5.02	***9.84***	*6.63*	***19.43***	***19.99***
10	2663.7					4.33	*9.57*
11	2726.9					*7.85*	6.15
12	2760.5					*7.62*	3.99
**13**	2975.7					*8.53*	***16.92***
**14**	3398.7					***11.52***	*9.68*
15	3524.8			*7.79*	4.61		
**16**	3531.1					***12.97***	7.14
**17**	3689.1					***11.83***	5.82
**18**	3696.8					***12.51***	4.98
19	3711.8					*7.68*	3.54
20	3723.8					7.41	*9.45*
**21**	3895.2					*9.38*	***20.37***
22	4101.3					3.78	*8.23*
**23**	4158.7					***14.8***	7.16
**24**	4174.8#	*7.99*	5.14	6.82	4.53	***9.62***	***12.81***
25	4184.5	*5.21*	*5.36*			*8.75*	4.67
26	4283.2			4.38	6.58		
**27**	4303.6					4.65	***11.64***

28	4487.0	2.55	*5.34*				
29	4532.9	4.47	*5.83*				
**30**	4608.5	***28.21***	***34.78***				
31	4992.9			*8.01*	6.33	*8.61*	*8.31*
**32**	5008.6			***23.39***	***16.91***	***32.28***	***38.78***
33	5025.2			*8.25*	*7.03*		
**34**	5034.4			*7.53*	***11.03***	***11.65***	***28.8***
**35**	5064.3#	***13.82***	***11.5***	***11.38***	***12.88***	6.23	*9.68*
**36**	5081.5	***11.73***	***9.62***	*7.18*	*8.97*		
**37**	5105.9#	***22.75***	***25.26***	***35.69***	***49.29***	5.82	***13.59***
**38**	5123.6			6.29	***9.96***		
39	5146.7			4.53	6.58		
40	5262.6	*6.51*	2.1				
41	6369.4					3.83	*7.19*
**42**	6444.1	***12.65***	***8.57***	*8.49*	5.75		
**43**	6657.6	*6.85*	***9.73***	4.11	*7.29*		
**44**	7734.1			***9.61***	*9.07*		
**45**	8154.2	*5.24*	*7.18*	*8.4*	***12.79***		
**46**	8203.3			5.3	*7.24*	*7.67*	***18.54***
**47**	8369.7	***8.15***	***11.12***				
**48**	8565.6			3.8	***10.96***		
49	8615.3			3.62	*8.02*	2.5	*11.46*
50	8972.8	3.66	*7.12*				
51	9046.9					3.14	*8.6*
**52**	9062.2	***8.34***	***9.02***	***8.94***	***9.58***		
53	9084.3	*5.32*	*5.64*				
**54**	9214.3	***50.56***	***53.13***	***12.72***	***15.15***		
**55**	9285.9	***8.61***	***10.48***				
56	11560.7	5.08	*8.35*				

§List of MS peaks used to distinguish *Glossina* species based on GA model analysis in ClinProTools. *Top twenty and top ten mass peaks per *Glossina* species are indicated in **bold** and ***bold+italic***. respectively. #MS peaks for which mass-to-charge ratio (m/z) were similar among the three body parts. Da: Daltons; m/z: mass to charge; a.u.: arbitrary unit.
